# The potential roles of exosomes in pancreatic cancer initiation and metastasis

**DOI:** 10.1186/s12943-020-01255-w

**Published:** 2020-09-02

**Authors:** Wei Sun, Ying Ren, Zaiming Lu, Xiangxuan Zhao

**Affiliations:** Department of Radiology, Shengjing Hospital of China Medical University, 36 Sanhao Street, Heping District, Shenyang, 110004 Liaoning China

**Keywords:** Exosome, Pancreatic cancer, Tumorigenesis, Metastasis, Tumor biomarker

## Abstract

Pancreatic cancer (PaCa) is an insidious and highly metastatic malignancy, with a 5-year survival rate of less than 5%. So far, the pathogenesis and progression mechanisms of PaCa have been poorly characterized. Exosomes correspond to a class of extracellular nanovesicles, produced by a broad range of human somatic and cancerous cells. These particular nanovesicles are mainly composed by proteins, genetic substances and lipids, which mediate signal transduction and material transport. A large number of studies have indicated that exosomes may play decisive roles in the occurrence and metastatic progression of PaCa. This article summarizes the specific functions of exosomes and their underlying molecular mechanisms in mediating the initiation and metastatic capability of PaCa.

## Background

Pancreatic cancer (PaCa) ranks among the most common and devastating digestive tract cancers worldwide. Besides early surgical resection, no effective regime against this aggressive malignancy has been discovered so far [1]. The 5-year overall survival rate of PaCa is considered less than 5% and the survival period of advanced PaCa is only 3–6 months [2]. Remarkably, the carcinogenesis of PaCa remains poorly characterized. The majority of PaCa cases (over 85%) are attributed to pancreatic ductal adenocarcinoma (PDAC) [3]. Some studies have shown that acinar-to-ductal metaplasia (ADM), induced by pancreatic injury, pancreatitis, or genetic toxicity, is one of the most typical events observed during PaCa development. ADM can further evolve into a variety of pancreatic intraepithelial neoplasias (PanINs), which are, to some extent, still reversible. Under the influence of many factors, PanINs eventually progress into PDAC [4]. ADM can be recovered after the elimination of oncogenic genetic insults or sustained environmental stress [5]. It has been shown that matrix metalloproteinase-7 (MMP-7) [6], NAD (+) - dependent protein deacetylase sirtuin-1 (SIRT1) [7], and polycomb compressor complex 1 (PRC1) [8] are involved in the regulation of ADM development. In addition, atypical flat duct lesions (AFDL) have often been considered as precancerous conditions of PaCa [9]. Recent studies have also indicated that mutations on *KRAS* [10], m (6) A demethylase gene *ALKBH5* [11], *PDL-1* (CD274), and various non-coding RNAs [12] may play key roles in modulating the occurrence and development of PaCa. Still, despite many reports describing a plethora of signal pathways involved in PaCa initiation and progression, the underlying mechanisms that orchestrate the development of this malignancy are poorly known or still under debate.

Exosomes, a general term for a particular class of nano-extracellular vesicles are produced by various stromal and transformed cells in the tumor microenvironment (TME) [13, 14]. Exosomes can be transported by a number of body fluids (i.e. blood, saliva, pancreatic duct fluid, cerebrospinal fluid and amniotic fluid) to distal tissues and organs, but, importantly, they can also function by autocrine and paracrine routes [15]. Exosomes can modulate the activation of various signaling pathways in target (recipient) cells. There is evidence showing that exosomes play crucial roles in the pathogenesis and evolution of many pancreas precancerous conditions, including diabetes mellitus (DM), pancreatitis, pancreatic fibrosis and other pancreatic-related disorders [16–18]. Exosomes can participate in promoting the transformation of various precancerous lesions to PaCa, including intraductal papillary malignant neoplasm (IPMN) and PanIN but, moreover, they may also play major roles in PaCa metastasis by inducing angiogenesis, cell migration, epithelial-mesenchymal transition (EMT), and apoptotic resistance [19, 20]. Our current work aims to discuss the biological significance of exosomes in PaCa carcinogenesis and metastasis.

## Exosome overview

### Characteristics of exosomes

**Fig. 1:**
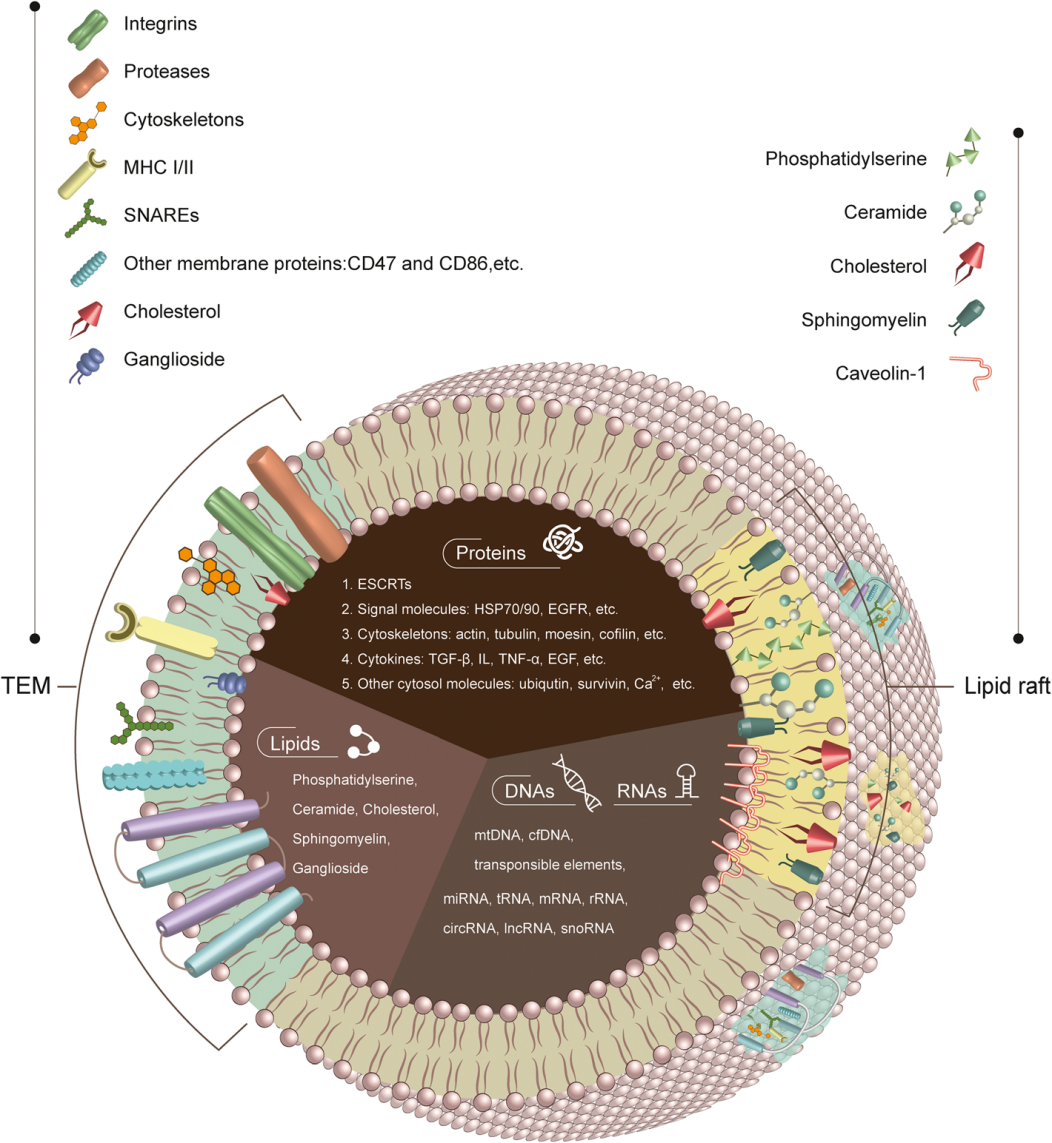
The main features of exosome

Exosomes mainly consist of spherical, disc or cup-shaped nanoparticles, coated by phospholipid bilayer, with a diameter of 40–150 nm (Fig. 1). These nano-structures typically contain proteins, nucleic acids, lipid molecules and other inorganic substances such as Ca^2+^ [13, 14]. Although exosomes can be generated by various types of cells, they all share similar structural proteins, including Rab GTPases, major histocompatibility complex class I and class II molecules (MHC I/II), Annexins, ALG-2 interacting protein X (ALIX), tumor susceptibility gene 101 protein (TSG101), flotillin (FLOT1), integrins, and tetraspanins (Tspans) [21].

Tspans belong to a 4-transmembrane protein family, mainly comprised of CD9, CD63, CD81, CD82, CD53, and CD37, which are ~ 100-fold more enriched in exosomes than in their parental cells [22]. Homodimers can be formed between Tspans, or heterocomplexes can be formed between Tspans and other proteins. Tspans may also couple with cholesterol and gangliosides to further generate distinct Tspan-enriched microdomains (TEMs). Depending on the cellular requirements, TEMs may selectively recruit membrane-related proteins, such as integrins, proteases and other related signal molecules, thus operating as a specific signal transduction platform [23].

The exosome surface also contains a variety of lipid raft microdomains such as caveolae that integrates caveolins as structural proteins that have a property to resist against detergents. These exosome microdomains (TEMS and caveolae) can transduce important signals, such as apoptosis and cell cycle arrest, via lipid molecules or proteins [24]. Exosomal lipids are comprised of cholesterol, ganglioside, sphingomyelin, (hexosyl) ceramide, phosphotidylserine, and phosphotidylethanolamine [25]. Exosomal nucleic acids mainly correspond to microRNAs (miRNAs/miRs), transfer RNAs (tRNAs), ribosomal RNAs (rRNAs), messenger RNAs (mRNAs), circular RNAs (circRNAs), long noncoding RNAs (lncRNAs), lincRNAs, cell free DNAs (cfDNAs) and mitochondrial DNAs (mtDNAs) [26]. According to the necessity of the cells, the protein content of certain exosomes may integrate particular signal molecules, including heat shock protein family proteins (such as HSP70/90), as well as cell membrane receptors (such as EGFR), cytokines, cytoskeletal molecules, and other cytosolic components such as ubiquitin, survivin, and Ca^2+^ [26]. At the same time, the lipid membrane components as well as the inner exosomal content may vary according to the exosome function and the type of exosome-producing cells.

### Exosome secretion and uptake

**Fig. 2:**
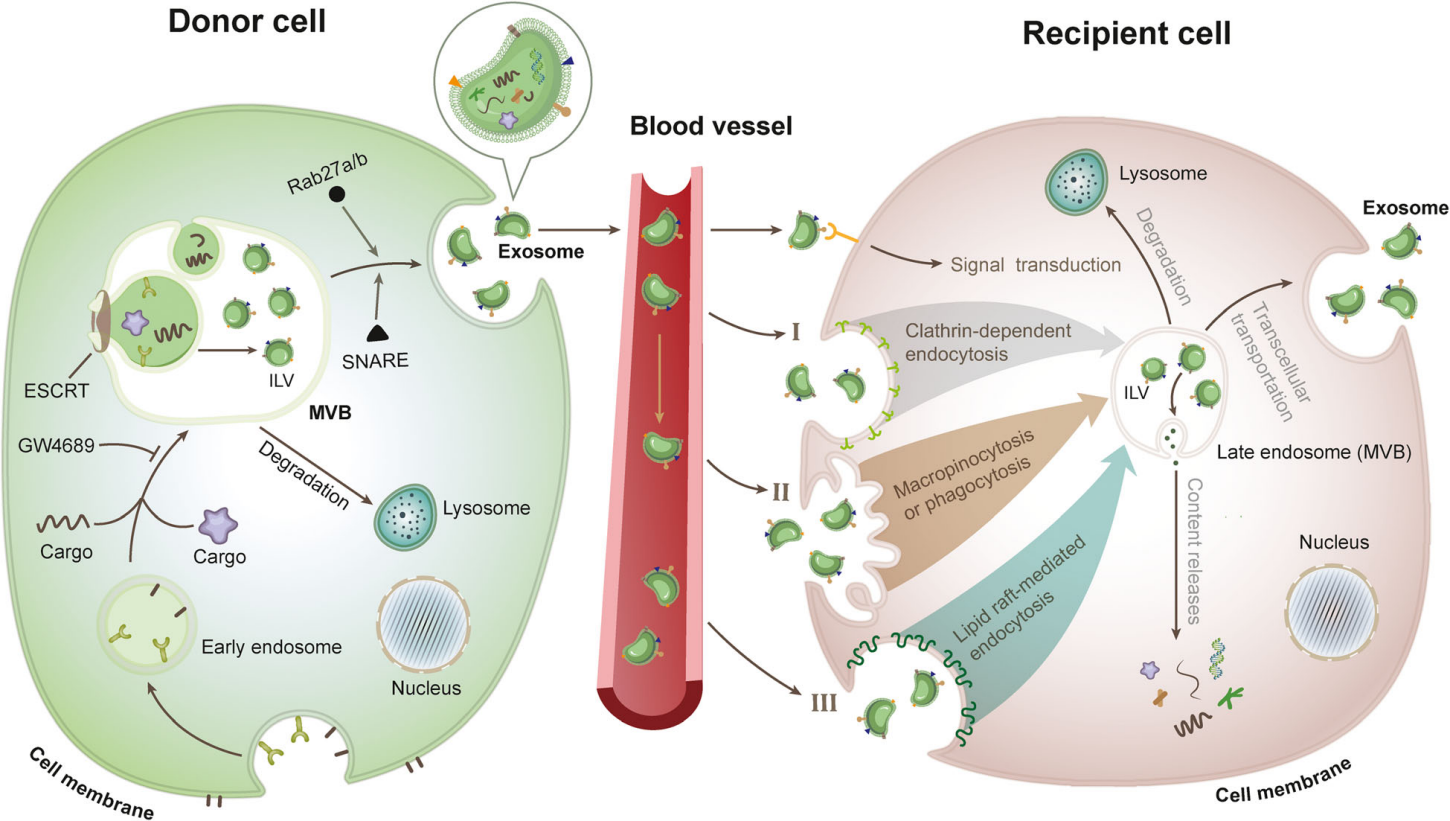
Exosome secretion and uptake. (i) Specific areas of the plasma membrane can invaginate along with cargo (es) to form an early endosome structure. Early endosomes then further continue to collect various cargoes from the cytosol, during which with the assistance of ESCRTs and other related proteins, the endosomal lipid membrane can wrap a range of wanted cargoes to form a variety of independently closed ILVs inside the late endosomes (MVBs). MVBs can move to and combine with lysosomes to digest their content and recycle them or, alternatively, fuse with the plasma membrane to secrete ILVs out of cells, thus forming exosomes. (ii) Exosomes can be transported by the body fluids, including blood, to reach their target or recipient cells. Exosomes function by directly transmitting signals upon binding to surface receptors of recipient cells, or being absorbed into the recipient cells through different endocytosis mechanisms to form endosomes again. Inside the target cells, exosomes can release their content into cytosol to fulfill various signaling transduction, be merged with lysosomes digest and recycle content, or re-fuse with the plasma membrane to accomplish the transcellular transportation

Exosomes originated from early endosomes, formed by invaginations along the cell membrane. Upon stimulation by specific signals, early endosomes continually collect a variable amount of cargoes from the cytosol, leading to their maturation into late endosomes, i.e. multivesicular bodies (MVBs) containing a large number of intraluminal vesicles (ILVs) [27] (Fig. 2). The formation of ILVs is mainly mediated by an endosome sorting complex required for transport (ESCRT) complex-dependent machinery [24]. MVBs can be transported to and fuse with the plasma membrane, culminating into the release of the internal ILV to the extracellular environment to form the so-called exosomes. MVBs can also fuse with lysosomes, by which their content is degraded and recycled for further utilization. The movement and fusion of MVB to the plasma membrane is largely regulated by specific signaling pathways. It has been shown that soluble N-ethylmaleimide-sensitive factor attachment protein receptors (SNAREs) and GTPase Rab family proteins (such as Rab27a/b) are implicated in the process of MVB fusion to the plasma membrane [28, 29]. Additionally, there is also evidence supporting that the formation of MVB does not depend on the existence of ESCRT complex. For instance, upon gene silencing of all *ESCRTs* (under the assistance of Tspans and ceramide), a large number of ILVs can still be formed in the MVB, which eventually move to the plasma membrane for further release of exosomes [30, 31]. GW4682, a small-molecule compound, can reduce the production of ESCRT-independent exosomes by inhibiting neutral sphingomyelinase 2 (nSMase2), which then generates ceramide from sphingomyelin [32]. Upon delivery of exosomes into recipient cells via body fluids (such as blood), these biological nanocarriers interact with cells by recognition and conjugation to membrane-bound receptors, therefore activating specific signal pathways in target cells. Alternatively, exosomes can be also internalized into target cells by different mechanisms, including clathrin-dependent, lipid raft (Caveolae/caveolin-1)-mediated endocytosis, and macropinocytosis/phagocytosis [33]. After entering the cells, MVBs (late endosomes) containing exosomes either fuse with lysosomes to recycle exosomal components, or release their content into the cytoplasm to further act as second messengers. Exosomes may also be secreted again from cells by mechanisms involving transcellular transport.

## Exosomes and PaCa initiation

Pancreatitis and diabetes mellitus (DM) are both considered non-malignant pancreatic diseases that may promote abnormal secretion of hormones, such as glucagon increase and insulin decrease, and tissue destruction that could eventually evolve into PaCa, if no preventive or therapeutic care is provided [34]. Furthermore, genetic mutations, obesity, viruses, and alcohol intake are also risk factors for PaCa occurrence [35, 36]. PaCa can develop from pre-malignant lesions, such as intraductal papillary mucinous neoplasm (IPMN) or pancreatic intraepithelial neoplasia (PanIN). Meanwhile, some key pro-oncogenic factors, including KRAS, CDKN2A (also known as p16/MTS1), SMAD4, and p53, have been reported be involved in the transition of pre-cancerous conditions to PaCa [37]. In this section, we discuss how exosomes are closely associated with the onset of pancreas precancerous diseases (Table 1).

**Table 1:** Potential exosomes biomarkers involved in pancreas precancerous diseases

Biomarkers	Sample	Receipt cell	Exosome function	Clinical significance	Refs
Calreticulin/Gp96/ORP150	Islet β-cells	DCs	Increase activation of APCs	T1DM	[38]
CCN2/miR-21	PSCs	PSCs	Stimulate the migration, proliferation, and division of PSCs as well as the collagen and fibronectin secretion	CP	[39]
FABP4 ↑	Serum	Not mentioned	Exacerbate insulin resistance and result in hyperglycemia	T2DM	[40]
GAD65/IA-2/Pro-insulin	Islet β-cells	DCs/T-cells	Decrease β-cell content and insulin secretion	T1DM	[38]
Klotho	MSCs	PACs	Block inflammatory responses and apoptosis	AP	[41]
RBP4	Adipocytes	Macrophages	Stimulate macrophages to secrete IL-6 or TNF-α	IR	[42]
TAAs ↑	Serum	B lymphocytes	Prevent B lymphocytes from recognizing PaCa cells	IS	[43–45]
MiR-16	SKMs	Islet β-cells	Inhibit β-cell proliferation	IR and T2DM	[46]
MiR-16-5p/574-5p/21-5p ↓	Serum	Not mentioned	Not mentioned	T1DM	[47]
MiR-30/133b/342 ↑	Urine	Adipocytes	Not mentioned	T2DM	[48]
MiR-106b-5p/222-3p-c	BMCs	Islet β-cells	Induce β-cells proliferation	T1DM	[49]
MiR-142-3p/142-5p/155	T lymphocytes	Islet β-cells	Induce apoptosis in β-cells	T1DM	[50]
MiR-146a/b/195/497	Islet tumor cells	Islet tumor/β-cells	Induce apoptosis in islet tumor cells and β-cells	T2DM	[51]
MiR-155/222/486	ADCs	Not mentioned	Not mentioned	DM	[52–54]
MiR-203	PaCa cells	DCs	inhibit the expression of TLR4, TNF-α, and IL-12	IS	[55]
MiR-212-3p	PaCa cells	DCs	Inhibit DCs from presenting antigens to T lymphocytes	IS	[56]
MiR-375 ↑	Serum	Not mentioned	Reduce insulin secretion and islet formation	T2DM	[57, 58]
MiR-1260a/494-3p	*PaCa cells	iPBMCs	Not mentioned	IS	[59]
lncRNA ENST/mRNA AEP/legumain	PaCa cells	DCs	Not mentioned	Not mentioned	[60]

↑, up-regulation; ↓, down-regulation

*Abbreviations*: *ADCs* adipose derived macrophages, *MSCs* mesenchymal stem cells, *BMCs* bone marrow cells, *iPBMCs* immunosuppressive peripheral blood mononuclear cells, *IR* insulin resistance, *IS* immune suppression, *lncRNA ENST* lncRNA ENST00000560647, *NF-κB* nuclear factor-κB, *PaCa cells SMAD4^−/−^ pancreatic cancer cells, *PAC* pancreatic acinar cells

### Exosomes and pancreatitis

Pancreatitis is usually divided into acute (AP), chronic (CP), and autoimmune (AIP) pancreatitis. AP relates to an acute auto-digestive inflammatory reaction of the pancreatic tissue, which can lead into pancreatic edema, hemorrhage and eventually necrosis, or parenchymal cell apoptosis. Moreover, AP is often accompanied by local or systemic complications and is considered highly lethal. AP-induced PaCa may occur independently of age, country (or geographic region), and etiology [61, 62]. In contrast, CP is correlated with various pro-inflammatory pancreatic secretion diseases and fibrosis, thus increasing the risk of PaCa by 12-fold [41, 63]. Generally, the risk of CP to evolve into PaCa within a 20-year range is ~ 5%, while the incidence of the disease increases annually thereafter [64]. Lastly, AIP is a distinct type of autoimmune pancreatic disease that is divided into diffuse and focal/segmental subtypes, whose correlation with PaCa is still unclear [65].

Increasing evidence has suggested that exosomes are involved in the occurrence, development of pancreatic inflammation (pancreatitis) as well as related carcinogenesis. Compared with healthy subjects, the number of circulating exosomes (cirExos) in the blood of AP patients (AP-cirExos) is significantly increased [66]. AP-cirExos appear to originate from liver or immune cells, and play a similar role as inflammatory factors by inducing a series of molecular reactions that may result in irreversible changes of interstitial fibrosis as well as parenchymal calcification of the pancreas [66]. AP-cirExos can break through the lung alveolar endothelial barrier and induce the transformation of macrophages from M2 to M1 to eventually promote an acute lung injury (ALI) [66]. Recent studies have indicates that AP-cirExos can induce the nucleotide binding oligomerization domain (NOD)-like receptor protein 3 (NLRP3)-dependent inflammasome activation and pyroptosis in alveolar macrophages of AP mouse model [67]. Moreover, AP can produce cirExos in ascites to further induce injury in other related tissues and organs via the hepatic or portal system [66]. In vivo studies using a rat model with taurocholate-induced AP have indicated that exosomes derived from pancreatitis-associated ascitic fluid (PAAF-Exos) and plasma exosomes (AP-cirExos) are two distinct populations. For instance, AP-cirExos exhibit much higher pro-inflammatory activity on macrophages than PAAF-Exos. One possible explanation for this difference is that the former contains some pro-inflammatory miRNAs such as miR-21/122/155, while the latter possesses high levels of histones and ribosomal proteins [68]. Klotho, an anti-aging protein is overexpressed in several malignancies, including breast cancer [69] and hepatocellular carcinoma [70], to induce apoptotic resistance and cell growth. Interestingly, exosomal Klotho derived from mesenchymal stem cells (MSCs) can attenuate the caerulein-induced activation of the nuclear factor-κB (NF-κB) signaling in pancreatic acinar cells, which potentially block inflammatory responses and apoptosis in AP. These observations suggest that exosomal Klotho could be utilized as a potential therapeutic target for AP [41].

Repeated episodes of AP can eventually evolve into CP. There are many pathogenic factors identified for CP, which is mainly characterized by a slow but progressive pancreatic tissue destruction and fibrosis that increase the risk of PaCa [71]. Indeed, CP can cause some substantial pancreatic tissue destruction as well as exocrine/endocrine insufficiency, which potentially activate resting pancreatic stellate cells (PSCs). The activated PSCs have a high proliferation capacity, which can be transformed into myofibroblasts [72, 73]. PSCs are able to communicate with PanINs and promote their progression [74, 75]. It has been shown that connective tissue growth factor 2 (CCN2/CTGF2), a fibrosis-related protein, can modulate a multitude of pancreatic cell functions, such as β-cell proliferation and fibronectin secretion in PSCs [71]. Clinical studies have also shown that CCN2 is highly expressed in PSCs from CP patients [39]. Mechanistic analyses have demonstrated that *CNN2* expression can be regulated by miR-21 [39]. Moreover, both CCN2 and miR-21 are found in exosomes produced by PSCs [39]. In vivo studies using a mouse alcoholic pancreatitis model have demonstrated that miR-21- and CCN2-positive exosomes can be retrieved by other PSCs in an autocrine or paracrine manner, therefore stimulating the migration, proliferation, division of PSCs as well as the collagen production [39]. These continuously activated PSCs-secreted collagens may precipitate and form extensive fibrotic lesions in pancreas. Clinically, a higher density stroma or the induction of fibrotic plaques by PSCs is typical characteristics of both CP and PaCa. These coincident features make these two pathological conditions hardly distinguishable [39].

Additionally, Zhao and coworkers have shown that rat pancreatic acinar cells can also produce exosomal miRNAs that are capable of activating pancreatitis-associated macrophages via MAPK signaling pathway [76]. However its physiological significance remains to be verified. Many viruses, including hepatitis viruses [77], human immunodeficiency virus [78], epstein-barr virus, [79] and coxsackie virus [80], can also cause pancreatitis. Among these viruses, coxsackie virus B3 (CVB3) infection has been reported to induce an increase on the levels of intracellular Ca^2+^ as well as cytoskeleton depolymerization in host cells, promoting the secretion of CVB3-positive exosomes that may propagate virus transmission upon uptake by non-infected cells. Of note, alcohol consumption has been referred to increase the incidence of CVB3-induced pancreatitis [81].

### Exosomes and diabetes mellitus (DM)

DM is a metabolic disease driven by genetic and/or environmental factors [82]. DM-mediated fat toxicity, chronic inflammation, and oxidative stress may modify the function of a number of cells, such as pancreatic α/β-cells, adipocytes, hepatocytes, and T-lymphocytes, and then increase the risk of PaCa [83]. There is also evidence indicating that the incidence of PaCa is significantly higher in DM patients than in the normal population, for which, one possible explanation is that insulin resistance and abnormal glucose metabolism can act as driving forces behind PaCa predisposition in DM patients [82]. In addition, blood glucose levels have been correlated with the prevalence of PaCa [67], besides serving as a predictor of tumor size and grading in PaCa patients [84].

Recent studies have shown that exosomes also mediate Type 1 DM (T1DM, mainly caused by autoimmune reaction) [85] and Type 2 DM (T2DM) [86]. Specifically, Delong and coworkers have found that exosomes appear to play crucial roles in islet autoimmune response in T1DM [87]. Antigen presenting cells (APCs)-derived exosomes contain large amounts of major histocompatibility complex (MHC), which are able to induce the T-cell immune response and activate B lymphocytes to initiate humoral immunity. These responses may specifically support the generation of an autoimmune attack towards islet β-cells, resulting in a decrease of β-cell content and eventually an increase in blood glucose levels and DM due to limited insulin secretion [38, 88]. Cianciaruso and colleagues have observed that exosomes released by both human and rat pancreatic islets contain β-cell autoantigens, such as glutamin acid decarboxylase 65 (GAD65), insulinoma-associated protein 2 (IA-2), and pro-insulin. These particular exosomes are also capable of entering and activating dendritic cells (DCs) [38]. Upon endoplasmic reticulum stress (ERS), T1DM-related cytokines, including IL-1β and IFN-γ, can induce β-cells to release exosomes containing calreticulin, heat shock protein Gp96, and oxygen-regulated protein 150 (ORP150) to further exacerbate T1DM-related autoimmune diseases [38]. Besides exosomal proteins, it has also been reported that certain miRNAs, including miR-142-3p, miR-142-5p, and miR-155, are specifically present in exosomes derived from murine and human T lymphocytes, which can promote apoptosis by up-regulating T1DM-related chemokines (i.e. Ccl2, Ccl7, and Cxcl10) in pancreatic β-cells [50]. Furthermore, Tsukita and colleagues have confirmed that miR-106b-5p- and miR-222-3p-containing exosomes, produced by bone marrow cells, can enter β cells to reduce *CIP/KIP* expression, resulting in β cell proliferation in vitro and in vivo [49]. Specifically exosomal miR-106b-5p and miR-222-3p can rescue streptozotocin-induced apoptosis of β cells in a mouse model, thus improving hyperglycemia [49]. Hence, exosomal miR-106b-5p and miR-222-3p may be exploited as potential therapeutic agents for DM. Finally, serum levels of exosomal miR-16-5p, miR-574-5p and miR-21-5p are significantly higher in healthy subjects when compared to T1DM patients [47], but their significance in PaCa initiation still require further validation.

T2DM is mainly caused by insulin resistance, decreased hormonal sensitivity, or reduced insulin production due to pancreatic β-cell dysfunction. Fatty acid-binding protein 4 (FABP4) is released by adipose tissues and may play a key role in the development of T2DM [89]. Upon enhancement of lipolysis in obese T2DM patients, the amounts of exosomal FABP4 in plasma also increase, exacerbating insulin resistance and then resulting in hyperglycemia and T2DM [40]. Additionally, miRNA-positive exosomes, produced by pancreatic islets, have been found to trigger elevated β-cell apoptosis in T2DM. For instance, Guay and colleagues have treated MIN6 pancreatic islet tumor cells with IFN-γ, TNF-α, and IL-1β, and found that exosomal miR-146a/b, miR-195, or miR-497, which are produced in response to treatment, can induce apoptosis in MIN6 cells as well as in mouse pancreatic islet cells [51]. MiR-375 plays an important role in maintaining glucose homeostasis [90]. It has been observed that overexpression of miR-375 suppresses glucose-induced insulin production in pancreatic β-cell lines and isolated primary β cells. In contrast, this overexpression can be abrogated by miR-375 inhibition or *myotrophin* gene silencing [91, 92]. Importantly, serum miR-375-3p-positive exosomes derived from the pancreas have been shown to mediate a reduction on insulin secretion and islet formation, which eventually results in T2DM [57, 58]. Still, miR-30, miR-133b, and miR-342 are apparently up-regulated in urinary exosomes isolated from T2DM patients [48]. Exosomes enriched with miRNAs, such as miR-155 [52], miR-222 [53], and miR-486 [54] (isolated from adipose-derived macrophages as well as MSCs and stem cells respectively), can act as potential modulators of DM. Nevertheless, the association between these exosomal miRNAs produced by adipose-derived cells and PaCa occurrence still requires further validation. Finally, miR-200 overexpression induces β-cell apoptosis, which may contribute to T2DM-related death [93]. Whether miR-200 is localized in exosomes also requires further evaluation.

### Exosomes-mediated immune suppression

Exosomes produced by cancer cells can support their escape of immune surveillance by inhibiting lymphocyte activation and survival, and inducing loss of function in lymphocytes [94]. Of all, DCs are the most important APCs in the human body, functioning in the immune system by inducing the expression of Toll-like receptors (TLRs) and producing various interleukins (ILs). Among TLRs, *TLR4* expression is particularly vital for the antitumor activity of DCs [95]. Exosomes produced by PaCa cells (PaCaExos) have been shown to induce immune suppression by deregulating DCs. For instance, miR-203-containing exosomes produced by PaCa cells are able to increase intracellular miR-203 levels and inhibit the expression of *TLR4*, *TNF-α*, and *IL-12* after being uptaken by DCs, and eventually induce their dysfuction [55]. It has also been shown that miR-212-3p-positive PaCaExos can specifically diminish the levels of MHC II transcription factor and regulatory factor x-associated protein (RFXAP) and, subsequently, inhibit DCs from presenting antigens to T lymphocytes [56]. Generally, exosomes containing tumor-associated antigens (TAAs), produced by cancer cells, can present MHC complexes to DCs for further processing and then activate the immune response by tumor-specific T lymphocytes [43, 44]. However, tumor cells are also shown to suppress both adaptive and innate antitumor responses via exosomes. As an example, the lipid membrane surface of cirExos from the plasma of PaCa patients contains a large amount of TAAs. These exosomes can specifically bind and harbor immunoglobulins in the plasma to prevent B lymphocytes from recognizing tumor cells, thereby enabling cancer cells to escape from the cytotoxic killing effects induced by immune cells [45]. In vivo studies have further confirmed that PaCaExos can effectively inhibit IL-2-mediated PI3K/Akt signal pathway in lymphocytes after being acquired by DCs and macrophages, which may eventually promote apoptosis [96]. Moreover, PaCaExos can increase the levels of lncRNA ENST00000560647 and asparaginyl endopeptidase (AEP/legumain) mRNA in DCs [60]. However, the precise function of these exosomes in immune escape needs to be further investigated.

Integration into a specific tumor microenvironment (TME) is considered a pre-requisite for cancer cell metastasis, proliferation, and survival. The formation of this microenvironment includes the transformation of immune cells (towards a immunosuppressive and pro-tumor phenotype) as well as fibroblast proliferation and increased fiber hyperplasia [97]. There is evidence demonstrating that, in PaCa TME, the amount of immunosuppressive T_reg_ cells, M2 polarized tumor-associated macrophages (M2TAM), and immature myeloid-derived suppressor cells (iMDSCs) are superior to those of immune effective CD8^+^ T cells, DCs, and M1 polarized TAMs [98, 99]. In this context, the immunosuppressive cells may help PaCa cells to escape immune surveillance. Notably, ~ 50% of PaCa cases lack expression of the tumor suppressor SMAD4 [100]. SMAD4-deficient PaCa cells can produce exosomes that contain miR-1260a and miR-494-3p. Upon uptake by immunosuppressive peripheral blood mononuclear cells (such as gMDSCs and mMDSCs), these miR-1260a and miR-494-3p-positive exosomes can promote cell proliferation and glycolysis, thereby creating an immunosuppressive TME [59]. There is also evidence that exosomes produced by PaCa cells in rats can be uptaken by various leukocytes, leading to the inhibition of cell proliferation and weakness of anti-apoptotic ability. Moreover, these particular PaCa exosomes are capable of inhibiting IL-12-induced T_h_ cell proliferation and abrogate the chemotactic migration of leukocytes to tumor sites [96], which contributes to TME formation.

### Exosomes-mediated metabolic disorders

Accumulating evidence has demonstrated that obesity caused by high fat/caloric diet, contributes to PaCa initiation, especially in Western countries [101–103]. It has been shown that exosomes derived from adipose tissue of obese B6 mice can induce the differentiation of peripheral blood monocytes into activated macrophages [42]. Functional analyses have demonstrated that adipose tissues are capable of producing retinol binding *protein* 4 (*RBP4*)-positive exosomes to stimulate activated macrophages that secrete IL-6 or TNF-α in a TLR4-dependent manner, thus eventually inducing insulin resistance [42]. In addition, the impact of palmitic acid, isolated from edible palm oil, on metabolic diseases like DM has attracted great attention from cancer biologists. For instance, it has been discovered that mice fed with a high palmitic acid diet (HPAD) exhibit a subset of metabolic symptoms including hyperglycemia, glucose intolerance, and insulin resistance. Furthermore, HPAD can promote myoblasts to produce more exosomes that, in turn, may induce skeletal muscle (SKM) cell differentiation [104, 105]. Specifically, HPAD is capable of stimulating SKM to produce miR-16-positive exosomes that can be uptaken by pancreatic β-cells, thus inhibiting β-cell proliferation. This cell growth inhibition is driven by the activation of intracellular Hedgehog-PTCH1 signaling pathway that may, ultimately, induce SKM insulin resistance and promote T2DM progression [46]. Therefore, SKM-specific exosomes exert both endocrine and paracrine effects that may lead to insulin resistance due to the reduction of β-cell content.

Obstructive sleep apnea (OSA) is known as a potential cause of intermittent hypoxia (IH). Remarkably, this condition appears to increase the risk of cancer, promote cancer progression, and also elevate cancer-related mortality. Specifically, IH is shown to promote tumor cell proliferation and angiogenesis by increasing the production of exosomes and regulating exosome content [106, 107]. Almendros and colleagues have demonstrated that chronic intermittent hypoxia (CIH) may increase the number of tumor-promoting exosomes in the blood. Compared with normal sleep populations (or treated OSA patients), serum exosomes derived from OSA patients can significantly promote the proliferation and migration of PaCa cells [108].

## Exosomes and PaCa metastasis

Extensive evidence has demonstrated that tumor-derived exosomes act as extracellular signalosomes, with roles involving TME remodeling [109]. On one hand, PaCaExo can transport nucleic acids, proteins, or lipids from parental to recipient cells, which induce pro-inflammatory activities, mediate vascular leakiness, suppresses immune response, regulate apoptotic resistance, and promote angiogenesis and proliferation, thereby facilitating tumor metastasis. On the other hand, PaCa-related cells such as cancer-associated fibroblasts (CAFs), tumor-associated macrophages (TAMs), cancer initiating cells (CICs) and PSCs generate exosomes that may promote growth, proliferation, drug resistance, EMT, migration, invasion and metastasis of PaCa cells (Table 2).

**Table 2:** Potential exosomes biomarkers involved in PaCa metastasis

Biomarkers	Sample	Target cell	Exosome function	Signaling pathway	Refs
CD44v6	hPaCa-CM	hPaCa cells	Enhance migration and invasion	Activate Wnt/β-Catenin pathway and increase PAI-1, MMP and TIM-1	[110]
CD151/Tspan8	rPaCa-CM	hPaCa cells	Promote EMT, migration and metastasis, and increase drug resistance	Increase expression of chemokine and receptor such as CXCR4 and EGFR3	[111, 112]
Claudin7	rPaCIC	hPaCa cells	Promote migration and invasion	Increase pAkt/Bcl-2/Bcl-XL /MDR1, promote matrix degradation, and reprogram SC and HPC	[113]
ICAM-1/AA	hPaCa-CM	Macrophage	Induce macrophage phenotype change and promote tumor growth	Increase VEGF, MCP-1, IL-6, IL-1β, MMP-9 and TNF-α	[114]
Lin28B	hPaCa-CM	hPaCa/PSC	Promote metastatic invasion	Activate Lin28B/let-7/HMGA2 /PDGFB axis	[115]
MIF	m/hPaCa-CM	KC/HSC	Promote the formation of the liver pre-metastatic niche	Up-regulate TGF-β expression and induce fibronectin secretion	[116]
Plectin	hPaCa-CM	hPaCa cells	Induce migration, proliferation and invasion	Not mentioned	[117]
Tspan8/106/49d	rPaCa-CM	EC	Induce proliferation, migration, sprouting and progenitors maturation of EC	Induce VEGF-independent angiogenesis	[118]
VEGF	hPaCa cells	hPaCa cells	Enhance tumor growth and angiogenesis	Activate VEGF signal pathway to stimulate angiogenesis and tumor growth	[119]
ZIP4	haPaCa-CM	hPaCa cells	Increase proliferation, migration, and invasion of non-metastatic PaCa cells	Not mentioned	[120]
miR-27a	hPaCa-CM	HMVEC	Promote cell survival and growth	Induce angiogenesis by inhibiting BTG2 expression	[121]
miR-222	CM/Serum	hPaCa cells	Enhance proliferation and invasion	Induce decrease, phosphorylation and redistribution of p27 via PPP2R2A/Akt axis	[122]
miR-301a-3p	hPaCa-CM	Macrophage	Enhance migration and invasion, and induce macrophage phenotype change	Activate PTEN/PI3K signaling pathway	[123]
miR-339-5p	mPaCa-CM	mPaCa cells	Enhance migration and invasion	Decrease expression of zinc finger protein ZNF689	[124]
miR-501-3p	Macrophage	hPaCa cells	Induce tumorigenesis and metastasis	Decrease TGFBR3 levels and activate TGF-β signaling	[125]
miR-1246/1290	hPaCa-CM	PSC	Promote PSC proliferation and pancreatic fibrosis	Induce Akt/ERK activation and increase α-SMA and procollagen type I C-peptide	[126]
mRNA-hTERT	hPaCa serum	PHFF	Induce proliferation and inhibit senescence	Enhance telomerase activity	[127]
circ-IARS	hPaCa-CM	EC/HUVEC	Promote angiogenesis and metastasis by enhancing endothelial monolayer permeability and inducing HUVEC growth	Down-regulate miR-122 and ZO-1, up-regulate RhoA, RhoA-GTP, and F-actin as well as promote focal adhesion	[128]
circ-PDE8A	hPaCa serum	hPaCa cells	Promote invasive growth	Activate MACC/MET/ERK/Akt axis	[129]

*Abbreviations*: *CM* culture medium, *EC* rat aortic epithelial cells, *haPaCa-CM* culture medium from hamster pancreatic cancer cells, *hPaCa-CM* culture medium from human pancreatic cancer cells, *HPC* hematopoietic cells, *m/hPaCa-CM* culture medium from mouse or human pancreatic cancer cells, *mPaCa cells* mouse pancreatic cancer cells, *PHFF* primary human foreskin fibroblasts, *rPaCa-CM* culture medium from rat pancreatic carcinoma cells, *rPaCIC* culture medium from rat pancreatic cancer initiating cells, *SC* stroma cells

### PaCa-produced exosomes and PaCa metastasis

#### PaCa exosomal proteins

PaCa-derived exosomes (PaCaExos) contain various protein molecules that can activate surrounding stromal cells and induce extracellular matrix (ECM) remodeling and neovascularization, thus establishing a TME to facilitate metastasis. In vivo studies using PaCa animal models have demonstrated that PaCaExos are rich in Tspan8, CD106, and CD49d [118]. Upon uptake by rat aortic epithelial cells (ECs), those PaCaExos activate the intracellular expression of *VWF* (von Willebrand factor), *TSPAN8*, *CXCL5*, *MIF* (migration inhibitory factor), *CCR1*, *VEGF* and *VEGFR2*, which lead to neovascularization by inducing EC proliferation, migration, sprouting, and progenitor maturation. Notably, Tspan8-enriched exosomes produced by PaCa cells can induce VEGF-independent angiogenesis around tumor tissues [118]. Costa Silva and colleagues have pointed out that PaCa can utilize exosomes to establish a pre-metastatic niche in distal organs, such as liver or lungs [130]. In this case, after exosomes derived from mouse PaCa cells were injected into healthy mice, they could be found in the liver [130]. Mechanistic analysis have shown that MIF-positive exosomes derived from PaCa cells can promote liver metastasis by increasing TGF-β expression in Kupffer cells (KCs) and also activating hepatic stellate cells (HSCs) to secret fibronectin [130]. Compared with healthy subjects or individuals with 5-year progression-free PaCa, PaCa patients with liver metastases usually exhibit elevated exosomal MIF levels in the serum [116]. Therefore, exosomal MIF may prominently function in the formation of the liver pre-metastatic niche. Additional evidence has demonstrated that PaCaExos that are positive for integrin αvβ5 usually reach the liver, whereas integrin α6β4- and α6β1-containing exosomes are transported to the lungs [116]. A recent study has demonstrated that protein kinase D1 (PRKD-1) expression is significantly downregulated in PaCa tissues when compared to non-tumor tissues [131]. Particularly, *PRKD-1* knockout can induce PaCa cells (Panc-1) to produce more exosomes. Moreover, PaCa xenograft mouse experiments have confirmed that *PRKD-1* knockout can increase the content of exosomes in the serum, thus promoting PaCa invasion. Mechanistic analysis has showed that alteration on PRKD-1 may stimulate PaCa cells to produce more integrin α6β4 positive exosomes to promote PaCa lung metastasis [131]. Furthermore, Li and colleagues have figured out that the formation of a pre-metastatic niche also requires the generation of new blood vessels [128]. Upon uptake by human umbilical vein endothelial cells (HUVECs), PaCaExos can activate Akt and ERK1/2 signaling pathways. This pathway activation promotes tube formation, by increasing Ras homolog gene family member A (RhoA) activity, as well as cytoskeleton remodeling, which drive a cell shrinkage due to the decreased expression of tight junction ligand protein Zonula occludens-1 (ZO-1), and also induce endothelial barrier dysfunction by enhancing local hyperpermeability [128]. In another study, Satake and colleagues have injected double fluorescence-labeled Mia-PaCa-2 cells into the spleen of nude mice and then demonstrated that PaCaExos reach the liver where they are uptaken by KCs, but also appear in the bone marrow and lung [132].

It has been shown that the knockout of *CD151* or *TSPAN8* expression (*CD151*^−/−^ or *TSPAN8*^−/−^, respectively) results in impaired metastasis of PaCa cells [111]. Remarkably, the re-introduction of regular PaCaExos into *CD151*^*−/−*^ or *TSPAN8*^*−/−*^ cells can restore metastasis. Functional analysis has shown that CD151- and Tspan8-postive exosomes are able to (i) activate the expression of EMT-related genes in PaCa cells, (ii) induce ECM remodeling by activating stromal cells, and (iii) up-regulate the expression of pro-inflammatory factors in hematopoietic cells [111]. Furthermore, lymphangiogenesis is impaired and the hypersensitivity reaction is delayed in *TSPAN8*^*−/−*^ mice, while angiogenesis is severely impaired in both *CD151*^*−/−*^ or *TSPAN8*^*−/−*^ mice. Still, metastasis of PaCa cells transplanted into either *TSPAN8*^*−/−*^ or *TSPAN8*^*−/−*^/*CD151*^*−/−*^ mice is effectively inhibited, suggesting that host Tspan8 or CD151 can significantly affect tumor progression [112]. Totally, PaCa exosomal CD151 and Tspan8 may promote matrix degradation and reprogramming of the stroma and hematopoietic cells, which are essential steps for PaCa metastasis. CD44 variant isoform 6 (CD44v6) is highly expressed in PaCa cells and can be integrated into exosomes [133]. Upon uptake of PaCa-derived CD44v6-positive exosomes by other PaCa cells, they activate Wnt/β-Catenin signaling and up-regulate the expression of plasminogen activator inhibitor 1 (PAI-1), MMP, and tissue inhibitor of metalloproteases 1 (TIM-1), thus enhancing PaCa cell migration and metastatsis [110]. Since CD44v6 can promote *TSPAN8* expression at the transcriptional level, *CD44v6* gene silencing effectively attenuates Tspan8-induced PaCa cell metastasis [134, 135]. Similarly, Jung and colleagues have observed that *CD44v6* gene knockout (*CD44v6*^*−/−*^) severely impairs PaCa cell metastasis. Co-treatment of CD44v6^*−/−*^ cells with soluble matrix (SM), produced by regular PaCa cells and PaCa-derived CD44v6-positive exosomes, can effectively restore the metastatic pattern of these cells, suggesting that PaCa may form a (pre-)metastatic niche microenvironment in distal metastasized organs by synergized effects derived of produced exosomes and other factors [136].

Myoferlin (MYOF) plays a crucial role in cell migration and invasion, as well as cell membrane endocytosis and vesicle transportation [137, 138]. It has been reported that MYOF can promote the migration and invasion of PaCa cells by regulating the mitochondrial structure and energy production [139, 140]. In PaCa cells, MYOF mediates the inclusion of VEGF into exosomes to promote tumor growth and angiogenesis. Accordingly, knockdown of *MYOF* expression can largely inhibit the growth and proliferation of PaCa cells [119]. Inhibition of MYOF function is also capable of reducing the volume of exosomes produced by PaCa cells as well as decreasing the levels of exosomal Rab7a and CD63. Although these exosomes with smaller volume are uptaken by human ECs, they fail to promote EC proliferation and migration, which eventually leads to inhibition of angiogenesis [118]. LIN28 is a 25-kDa RNA-binding protein that has been shown to promote PaCa growth and metastasis by inhibiting the biogenesis of a group of microRNAs, including let-7. The NAD(+)-dependent histone deacetylase sirtuin 6 (SIRT6) is able to induce PaCa growth inhibition by reducing LIN28 in PaCa cells [141]. Liver metastasis studies using PaCa tumor-bearing mice have demonstrated that LIN28B-positive exosomes produced by PaCa cells may reach target cells and activate the LIN28B/let-7/HMGA2/PDGFB signaling axis to further promote PaCa metastasis after injection via caudal vein [115].

Claudin 7 (Cld7) is a key structural protein present in tight junctions that interconnect cells [142]. It has also been shown that Cld7 can be distributed beyond TJ sites. For instance, palmitoylated Cld7 (Palm-Cld7) is localized in glycolipid-enriched membrane microdomains (GEMM) [113]. Cld7 in tight junction (TJ-Cld7) is shown to regulate the entry of related proteins into PaCaExos and affect the function of exosomes derived from CICs (CIC-Exos) by modulating the composition of exosomal transporters and lipid metabolites, while Palm-Cld7-positive exosomes have the capability of regulating cell migration [113]. Importantly, Kyuno and colleagues have found that murine pancreatic cancer initiating cells (PaCICs) can produce Cld7-positive exosomes which are capable of inducing re-programming of non-metastatic cancer cells to further increase their invasiveness [113]. Another PaCa-derived Wnt5β-positive exosomes have been reported to enter and activate the Wnt5β signaling in other cancer cells lines such as PaCa, A549 and Caco-2, where they stimulate migration and proliferation. *Wnt5β* knockout and *TSG101* silencing can both abrogate the exosomal Wnt5β-dependent PaCa cell proliferation and migration [143]. Under normal physiological conditions, plectin is usually localized in the cytoplasm where it functions as a scaffolding protein. Plectin is expressed in PaCa, but usually undetectable in non-PaCa tissues [144]. In PaCa cells, integrin β4 mediates the transfer of overexpressed plectin into exosomes, eventually leading to the proliferation, migration, and invasion of these cells [117].

Zinc transporter ZIP4-positive exosomes, produced by highly metastatic PaCa cells, can stimulate the proliferation, migration, and invasion of non-metastatic PaCa cells [145]. Accordingly, exosomal ZIP4 from the serum of PaCa patients can be used as a diagnostic marker for cancer progression [145]. Compared with exosomes derived from human pancreatic ductal epithelial cells (HPDE), exposure of non-tumorigenic cells to PaCaExos potentially induces transformation as well as tumorigenesis in vivo of non-malignant cells [120]. Functional analysis have indicated that PaCaExos are capable of inducing random gene mutations in recipient cells, while only certain cell populations with PaCaExo-induced mutations can undergo transformation and, eventually, become tumors. Considering the stochastic nature of mutations, the mechanism of PaCaExo-induced tumorigenesis in transformed cells may differ from each other [120]. Specifically, it has been reported that mutated DNA segments from *KRAS*, *CDKN2A*, *P53*, and *SMAD4* can be internalized into PaCaExos. Thus, these exosomes may effectively promote the transformation of normal cells as well as subsequent tumor formation [146].

#### PaCa exosomal nucleic acids

It has been shown that miR-27a is overexpressed in cancer tissues from PaCa patients as well as PaCa cell lines [147]. PaCa-derived exosomes containing miR-27a can induce proliferation, invasion and angiogenesis in human microvascular endothelial cells (HMVECs) by suppressing B-cell translocation gene 2 (BTG2), which promotes PaCa cell survival and growth [121]. In contrast, in vivo studies using PaCa animal models have demonstrated miR-339-5p can inhibit cell invasion and migration by down-regulating the expression the zinc finger protein ZNF689. MiR-339-5p levels are significantly reduced in exosomes from highly metastatic PaCa cells. Accordingly, the exogenous introduction of miR-339-5p can effectively inhibit PaCa migration and invasion [124]. MiR-222 is overexpressed in highly invasive PaCa cells, where it is assimilated into exosomes. Upon uptake by poorly invasive PaCa cells, exosomal miR-222 is then released to further decrease the expression, phosphorylation, and nuclear exit of p27 via the PPP2R2A/Akt axis, which ultimately promotes the proliferation and invasion of respective cancer cells [122]. Moreover, abnormal ECM accumulation and blood vessel depletion in the TME can cause high desmoplasia and extreme hypoxia in PaCa tissues, which in turn stimulates cancer cells to ensure their survival by offsetting the hypoxic/ischemic environment via compensatory metabolic mechanisms that promote PaCa progression and apoptosis resistance [148]. The hypoxic environment inside the tumor, which is caused by rapid cell growth, can stimulate the production exosomal miR-301a-3p in PaCa cells [123]. After being acquired by other PaCa cells, miR-301a-3p-positive PaCaExos can promote the metastatic ability and invasiveness of these cancer cells. Upon uptake by macrophages, miR-301a-3p can also induce HIF1α/2α-dependent M2 phenotype transformation due to the activation of PTEN/PI3K signaling cascade [123]. Hypoxia has been shown to stimulate PaCa cells to generate more of small-volume exosomes via HIF1α, which increases the survival, proliferation, and metastasis of PaCa cells [149]. Additionally, exosomal miR-1246 has been found in the serum from patients with breast and prostate cancers [150, 151]. High levels of miR-1246 have been associated with GEM-resistance in PaCa cells, which can promote PaCa metastasis, invasion, cancer stemness, and angiogenesis due to the inhibition of CCNG2 expression [152]. However, it still remains unclear whether miR-1246 can enter exosomes to affect the chemo-resistance in pancreatic cancer.

Besides the above distinct miRNAs, cancer tissues originated from PaCa patients have presented high levels of circular RNA IARS (circ-IARS) [128]. Exosomal circ-IARS produced by PaCa cells can promote cancer metastasis by increasing endothelial monolayer permeability and activating HUVECs to enhance angiogenesis. Mechanistic analyses have revealed that circ-IARS-positive exosomes may contribute to tumor invasion by (i) down-regulating miR-122 and ZO-1, (ii) up-regulating RhoA, RhoA-GTP, and F-actin and (iii) promoting focal adhesion. The high expression of circ-IARS has been positively correlated with liver metastasis, vascular invasion, and tumor-node-metastasis (TNM) of PaCa [128]. Li and colleagues have verified that metastatic PaCa cells in the liver present high levels of circular RNA PDE8A (circ-PDE8A). Serum circ-PDE8A-positive exosomes can induce invasive growth of PaCa cells by counteracting with miR-338 to activate the MACC/MET/ERK/Akt signaling axis [129]. Therefore, exosomal circ-PDE8A may be considered as a putative marker to predict PaCa metastatic progression. Additionally, exosomes from the serum of PaCa patients may also contain human telomerase reverse transcriptase (*hTERT*) mRNA [127]. PaCa-derived exosomes that are *hTERT* mRNA-positive can induce the transformation of non-malignant pancreatic fibroblasts (PF) into cells with high telomerase activity, thus stimulating cell proliferation and delaying aging [127].

### Exosomes produced by other cells and PaCa metastasis

#### CAFs

Cancer-associated fibroblasts (CAFs) that comprise main constituent cells of PaCa are essential for establishing the TME [153]. In TME, CAFs are able to regulate various behaviors and characteristics of PaCa cells, including epithelial-mesenchymal transition (EMT), proliferation, migration, invasion, metabolic transformation, and chemotherapy resistance [154, 155]. It has been shown that CAF-derived exosomes can reprogram the energy metabolism and up-regulate mitochondrial oxidation in PaCa cells, which induces glycolysis and glutamine-dependent reductive carboxylation to provides amino acids, fatty acids, as well as tricarboxylic acid cycle (TAC) intermediates for PaCa cells that are nutritionally deficient, thereby promoting the survival and growth of these cancer cells [156]. CAF-derived exosomes induced by gemcitabine (GEM) can activate miR-146a and the Snail signaling cascade in PaCa cells, thus promoting survival, proliferation, and drug resistance [157]. Interestingly, extracellular vesicles (EVs) containing annexin A6/LDL receptor-related protein 1/thrombospondin 1 (ANXA6/LRP1/TSP1) are solely present in the serum from PaCa patients. ANXA6/LRP1/TSP1-positive EVs are only produced by CAFs from PaCa patients, which are essential for liver metastasis [158]. Nevertheless, further studies are required to validate whether the ANXA6/LRP1/TSP1 complex may enter exosomes to support the aggressiveness of PaCa.

#### TAMs

Tumor-associated macrophages (TAMs), a class of TME-infiltrating macrophages, are capable of promoting the radio/chemotherapy resistance, angiogenesis, migration, invasion and metastasis of tumor cells [159]. It has been demonstrated that TAM-producing exosomes can promote PaCa metastasis and progression. For instance, exosomal miR-501-3p derived from M2 macrophages is able to inhibit *TGFBR3* expression and then activate TGF-β signaling, therefore inducing the formation and metastasis of PaCa xenografts in nude mice [125]. Analysis of clinical PaCa tissue specimens has indicated that miR-501-3p is also highly expressed in PaCa tissues. Exosomes produced by different types of PaCa cells (AsPC-1, BxPC-3, Panc-1, and MiaPaCa-2) contain distinct membrane proteins and lipid components that may affect the communication between PaCa cells and TAMs. For instance, exosomes produced by the ascites-derived human PDAC cell line AsPC-1 (AsPC-1-Exos) contain a large amount of ICAM-1 and arachidonic acid (AA). Since ICAM-1 can recognize and bind to CD11c on the surface of THP-1-derived macrophages (TDMs), AsPC-1-Exos can be uptaken rapidly by TDMs. The hydrolysis of AA in AsPC-1-Exos, catalyzed by phospholipase A2, has been shown to effectively reduce the fusion of AsPC-1-Exos with TDMs [114]. Treating non-polarized M0 macrophages with AsPC-1-Exos can induce M0 macrophages to transition into immunosuppressive M2 macrophages. Moreover, AsPC-1-Exos can simulate TDMs to secrete a subset of cytokines, such as VEGF, MCP-1, IL-6, IL-1β, MMP-9, and TNF-α, thereby promoting the growth and progression of PaCa [114].

#### CICs

Cancer initiating cells (CICs) play a major role in the initiation of cancer cell migration and metastasis [160]. PaCa-related CICs (PaCICs) can induce tumor stroma reorganization, stimulate angiogenesis, and promote hematopoietic cells to generate immunosuppressive cells via the production of exosomes (PaCIC-Exos), which eventually create a (pre-)metastatic niche in the distal metastatic organ [161, 162]. PaCIC-Exos can transfer certain characteristics of CICs to non-CICs, therefore inducing their reprogramming and promoting transformation characteristics such as anchorage-independent growth, apoptosis resistance, migration, and invasion, until they are phenotypically modified into CICs [163, 164]. In addition, PaCIC-Exos can be uptaken by non-CICs and then increase the levels of p-Akt, Bcl-2, Bcl-X_L_, and MDR1, which potentially leads into the induction of metastatic growth, cisplatin resistance, EMT, migration, and invasion. Intravenous injection of Tspan8 antibody (CO029) has been shown to properly inhibit the drug resistance of non-CICs induced by CICs via exosomes [165], suggesting its use as a potential therapeutic target for PaCa.

In PaCa cells, the expression of Tspan8 and other CIC marker proteins, such as integrin α6β1, CD104, EpCAM, CXCR4, and CD44v6, are mutually regulated. It has been demonstrated that knockdown of *CD44v6* expression is capable of decreasing the invasiveness of PaCICs [165]. In contrast, CD151-positive exosomes produced by CICs can induce EMT and migration of PaCa cells [111]. CIC-produced Cld7-positive exosomes exhibit some activity to promote non-metastatic PaCa dissemination and metastatic growth, by increasing cell migration, invasion, and angiogenesis. Still, Cld7-positive exosomes do not have an apparent impact in apoptotic resistance, proliferation and EMT of tumor cells [166]. Functional studies have demonstrated that CIC-Exos can rescue the defects caused by Cld7 loss in *CLD7*^*−/−*^ PaCa cells, by activating integrin signaling pathway, proteases (such as uPA) and lymphangiogenic receptor (for instance, VEGFR3). Interestingly, the ability of CIC-Exos to promote tumor progression by activating receptor tyrosine kinase (RTK) can be blocked by the RTK inhibitor Sunitinib, indicating that RTK inhibition could be serves as a therapeutic approach in PaCa [166]. Furthermore, incubation of rat PaCa cells with Tspan8-positive exosomes carrying Cld7-specific miRNA may cause *CLD7* gene silencing in vitro. Tspan8 can significantly enhance the targeting of exosomal Cld7-related miRNAs to PaCa sites, leading to decreased Cld7 levels and further reduction on the expression of other CIC markers and Notch1, which suppress tumor cell growth, motility, and invasion [167]. Therefore, these modified exosomes may effectively load and carry nucleic acid fragments to tumor sites and help inhibit tumor progression.

#### PSCs

Pancreatic stellate cells (PSCs) play crucial roles in chronic pancreatitis and pancreatic fibrosis [72]. These cells are capable of interacting with tumor and surrounding stromal cells (such as immune and endothelial cells) to respectively promote cell growth and distant metastasis [168]. Exosomes produced by PSCs can stimulate PaCa cells to express a number of chemokines, including CCL20, CXCL1/2, PDZK1IP1, SAA1/2, SMCR7L, and ZNF619, which in turn promote the proliferation and migration of PaCa cells [169]. PaCa cell lines including Panc-1 and SUIT-2 can produce miR-1246 and miR-1290-positive exosomes to induce cell proliferation and migration of PSCs by up-regulating the expression of α-smooth muscle actin (α-SMA/ACTA2), increasing procollagen type I C-peptide production, and activating ERK/Akt signaling cascades [126]. Activated PSCs subsequently produce exosomes containing CD9, CCN2 and miR-21, which exacerbate tumor tissue fibrosis by stimulating other PSCs to secret and deposit more collagen [39]. Thus, PSCs communicate with PaCa cells via exosomes in the TME, which is essential for PaCa progression.

## Conclusion and perspectives

**Fig. 3:**
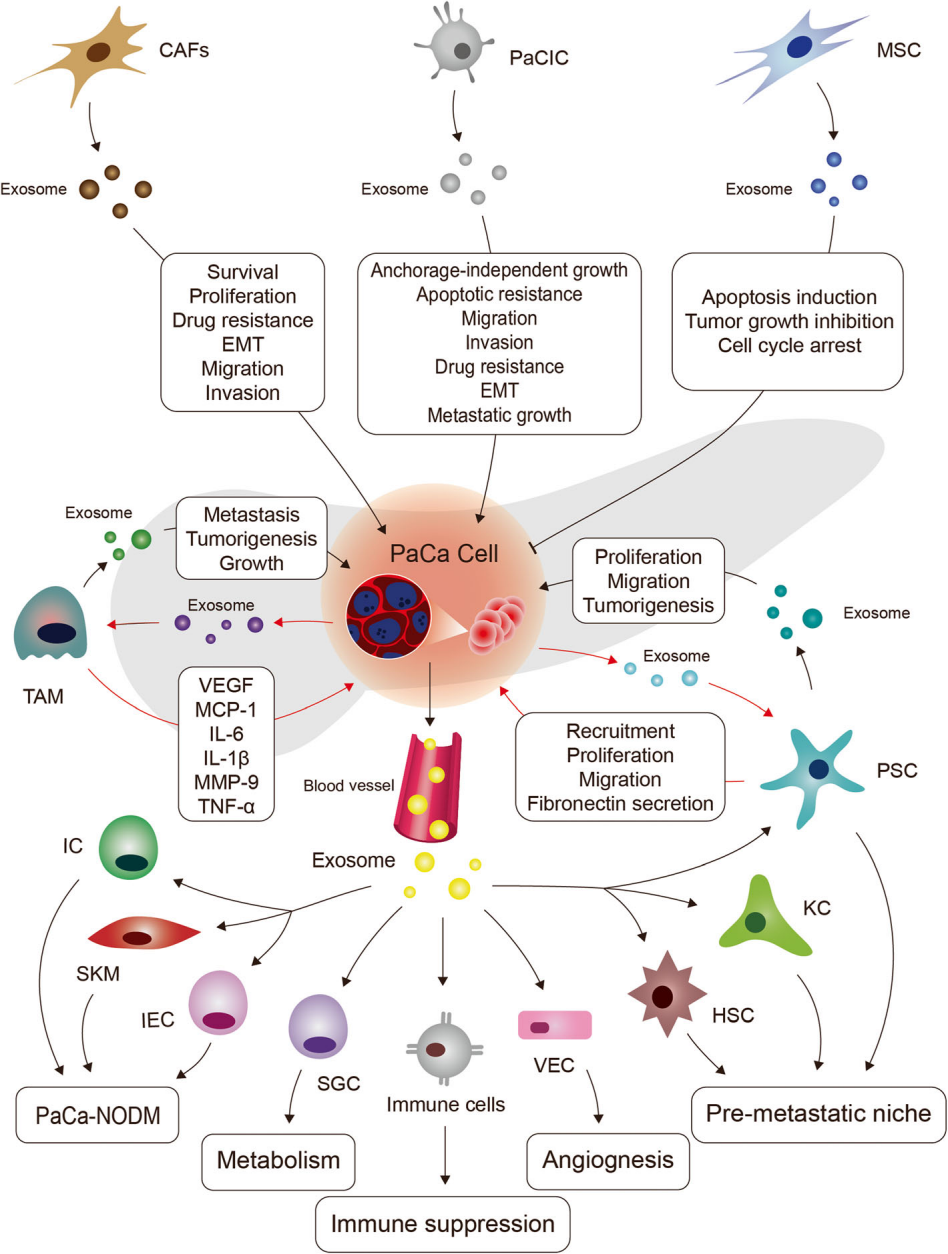
Crosstalk between PaCa cells and PaCa-related cells. PaCa cells can interact with a variety of PaCa-related cells to fulfill their metastatic progress. On one hand, PaCa-related cells (CAFs, TAMs, PSCs, and PaCaCICs) generate exosomes that can promote PaCa cell survival, proliferation, apoptotic resistance, drug resistance, EMT, migration and metastatic invasion. Notably, MSCs can produce exosomes that induce apoptosis, cell cycle arrest and growth inhibition in PaCa cells. On the other hand, PaCa cells can also produce exosomes to stimulate various related cells to secrete various cytokines or exosomes, which may create a facilitating tumor microenvironment for their own survival and metastasis. Specifically, PaCa-derived exosomes can stimulate TAMs to produce many cytokines, including VEGF, which in turn can induce a variety of metastatic characterization changes such as EMT in PaCa cells. PaCa -derived exosomes can recruit and stimulate PSCs to proliferate, migrate and secrete more fibronectin, thereby creating a metastasis microenvironment. PaCa cells may additionally produce exosomes to deregulate the body metabolism, impairing the functions of ICs, IECs, SGCs and SKMs. PaCa-derived exosomes can stimulate the proliferation and migration of VECs, thus forming new blood vessels, inducing KC and HSC activation to form a distant metastasis microenvironment in the liver, as well as targeting immune cells (including DCs) to promote immunosuppression

Our current study discusses the roles of exosomes towards PaCa initiation and metastatic progression. To date, most of the published studies have started to uncover the notion that exosomes exert their biological function in definition of PaCa pathogenesis and may serve as culprits behind PaCa metastasis. On one hand, exosomes mediate pancreas precancerous diseases caused by various conditions, including diabetes, inflammation and viral infections, which can promote and accelerate their transformation into PaCa. This suggests that targeting relevant exosomes may help preventing PaCa from precancerous stages, restoring the abnormal pancreas to a healthy state, and developing earlier diagnostic methods. On the other hand, key nodal studies, such as the identification of exosomes and their detailed molecular mechanisms involved in ADM or AFDL are still limited and may need to be strengthened and further explored. During recent years, it has been seminal to elucidate whether some newly discovered important regulatory factors, such as m (6) A modified regulatory factors, PDL-1, and non-coding RNAs, participate in the development of PaCa through exosomes-mediated signaling cascades. More importantly, a variety of PaCa-related cells, including PaCaCICs, CAFs, PSCs, MSCs, DCs, HSCs, TAMs, and PaCa-NODM-related cells, can crosstalk with PaCa cells through exosomes (Fig. 3). This intercommunication can not only establish a facilitating microenvironment for PaCa metastasis by generating exosomes around the tumor or in the distant organs, but can also render particular properties to PaCa cells such as apoptosis resistance, migration, EMT, and ultimately metastasis.

It is worth noting that, along with the occurrence and metastasis of PaCa, the human body also drives some biological reactions, including immune response to limit PaCa progression, even though these antitumor effects may not be dominant. For instance, the abovementioned MSCs can produce some special exosomes after differentiation into various subgroups, which induce apoptosis, cycle arrest and growth inhibition of PaCa cells. In addition, studies have confirmed that these endogenous exosomes may carry various substances without causing an immune response in the body, suggesting their utilization as putative drug carriers [170]. As an example, it has been found that exosomes derived from macrophages may carry chemotherapeutic drugs (doxorubicin), which are toxic to PaCa cells [171]. Interestingly, exosomes generated by PaCa cells also contain some tumor suppressor components, which may exert anti-cancer activity by inducing apoptosis and inhibiting proliferation of related cancer cells [172]. These findings have revealed the potential therapeutic value of exosomes. Therefore, to exploit these favorable variables may contribute to the diagnosis and treatment of PaCa in the near future. Nevertheless, it must be said that studies focusing on PaCa-related exosomes are still under progression. Some technical limitations, such as an effective exosome delivery, high pure and bulk exosome preparation with standard protocol, and exosome target specificity, are the biggest challenges and still need to be properly considered before any diagnostic or therapeutic applications are established.

## Data Availability

All data and information in this review can be found in the reference list.

## Abbreviations

ADM: Acinar-to-ductal metaplasia
AIP: Autoimmune pancreatitis
AP: Acute pancreatitis
APCs: Antigen presenting cells
AP-cirExos: Circulating exosomes from AP patients
CAFs: Cancer-associated fibroblasts
CD44v6: CD44 variant isoform 6
CICs: Cancer initiating cells
CIC-Exos: Exosomes derived from CICs
CTGF2/CCN2: Connective tissue growth factor 2
CP: Chronic pancreatitis
cirExos: Circulating exosomes
Cld7: Claudin 7
CVB3: Coxsackie virus B3
DM: Diabetes mellitus
ECM: Extracellular matrix
ECs: Epithelial cells
EMT: Epithelial-mesenchymal transition
ESCRT: Endosome sorting complex required for transport
FABP4: Fatty acid-binding protein 4
GEM: Gemcitabine
HPAD: High palmitic acid diet
HUVECs: Human umbilical vein endothelial cells
IH: Intermittent hypoxia
ILs: Interleukins
IPMN: Intraductal papillary malignant neoplasm
ILVs: Intraluminal vesicles
MHC: Major histocompatibility complex
MIF: Migration inhibitory factor
miRNAs/miRs: microRNAs
MYOF: Myoferlin
MSCs: Mesenchymal stem cells
MVBs: Multivesicular bodies
OSA: Obstructive sleep apnea
PAAF-Exos: Pancreatitis-associated ascitic fluid
PaCa: Pancreatic cancer
PaCICs: Pancreatic cancer initiating cells
PaCaExos: Exosomes from PaCa cells
Palm-Cld7: Palmitoylated Cld7
PanINs: Pancreatic intraepithelial neoplasias
PDAC: Pancreatic ductal adenocarcinoma
PRKD-1: Protein kinase D1
PSCs: Pancreatic stellate cells
RTK: Receptor tyrosine kinase
SKM: Skeletal muscle
TAAs: Tumor-associated antigens
TJ-Cld7: Cld7 in tight junctions (TJ)
TAMs: Tumor-associated macrophages
TME: Tumor microenvironment
TEMs: Tspan-enriched microdomains
TLRs: Toll-like receptors
T1DM: Type 1 DM
T2DM: Type 2 DM
